# Multisite Colonization Ecology of Multidrug-Resistant and Commensal Bacteria Among Cardiovascular Outpatients: A Cross-Sectional Study from Cairo, Egypt

**DOI:** 10.3390/antibiotics15090823

**Published:** 2026-08-25

**Authors:** Yasmin Abdelkhalek, Dina H. Amin, Hassan M. Gebreel, Hayam A. E. Sayed, Ahmed M. H. Kamel

**Affiliations:** 1 Department of Microbiology, Faculty of Science, Ain Shams University, Cairo 11566, Egypt; dina.hatem@sci.asu.edu.eg (D.H.A.); hassangebreel@sci.asu.edu.eg (H.M.G.); hayam84@sci.asu.edu.eg (H.A.E.S.); 2 Department of Cardiology, Faculty of Medicine, Ain Shams University, Cairo 11566, Egypt; am.hassan88@gmail.com

**Keywords:** antimicrobial resistance, bacterial colonization, multisite colonization, polymicrobial colonization, commensal flora on selective media, cardiovascular outpatients

## Abstract

Background/Objectives: Bacterial colonization is an under-recognized component of antimicrobial resistance (AMR), particularly among cardiovascular outpatients who frequently interact with healthcare services. While surveillance has traditionally focused on multidrug-resistant (MDR) pathogens causing clinical infection, the ecological characteristics of colonization including multisite carriage, polymicrobial communities, and commensal or low-virulence organisms recovered on antimicrobial-containing media remain poorly defined. This study investigated the burden and ecological patterns of colonization by organisms with reduced antimicrobial susceptibility in cardiovascular outpatients. Methods: In this cross-sectional study, 36 adult cardiovascular outpatients were screened using sputum, nasal, and skin specimens (108 samples) on antimicrobial-supplemented selective media. Isolates were identified by MALDI-TOF mass spectrometry, susceptibility was determined using the VITEK 2 system, and colonization patterns were analyzed using exact statistical methods. Results: Colonization by organisms with reduced antimicrobial susceptibility was detected in 61.1% of patients (95% CI, 44.9–75.2), and at least one validated MDR pathogen in 52.8% (95% CI, 37.0–68.0). Sputum showed the highest colonization prevalence. Multisite colonization occurred in 72.7% of colonized patients, with 40.9% positive at all three anatomical sites. Polymicrobial colonization was common (mean 1.9 species per colonized patient). Resistant commensal bacteria including *Neisseria flavescens*, *Neisseria mucosa*, *Moraxella catarrhalis*, and *Lacticaseibacillus casei* were recovered alongside recognized pathogens and showed reduced susceptibility across multiple antibiotic classes. Colonization showed significant concordance across anatomical sites, indicating a whole-body colonization state rather than a series of independent site events. Prior hospitalization and concurrent antibiotic therapy were significantly associated with colonization, whereas prior percutaneous coronary intervention and individual comorbidities were not. Conclusions: Cardiovascular outpatients frequently harbour complex bacterial communities characterized by multisite colonization, polymicrobial carriage, commensal and low-virulence organisms recovered on selective media, and MDR pathogens. These findings highlight bacterial colonization as a potential community reservoir of AMR and support targeted pre-procedural screening together with community antimicrobial stewardship. As a single-centre, cross-sectional study of 36 patients, these results are exploratory and hypothesis-generating and require confirmation in larger, multicentre cohorts.

## 1. Introduction

Long before an organism causes disease, it colonizes. Asymptomatic carriage of multidrug-resistant (MDR) bacteria is defined, following Magiorakos et al. [1], as acquired non-susceptibility to at least one agent in three or more antimicrobial categories on the skin, in the nares and in the respiratory tract, which constitutes the hidden compartment of the antimicrobial resistance (AMR) problem: it precedes infection, seeds transmission, and is far less often surveyed than the clinical isolates that dominate resistance statistics. Globally, bacterial AMR was linked to more than a million deaths in 2019 and is projected to account for tens of millions of deaths by the middle of the century [2,3], yet most surveillance, especially in low- and middle-income countries, still samples symptomatic, hospitalized or intensive-care patients rather than the colonized ambulatory population from which many of these organisms ultimately arise [4,5].

First, antimicrobial resistance is not restricted to recognized pathogens. Commensal and low-virulence bacteria recovered under antimicrobial selective pressure are increasingly recognized as overlooked components of antimicrobial resistance ecology. Despite often being excluded from routine surveillance or dismissed as contaminants, evidence suggests that they may contribute to the maintenance and dissemination of antimicrobial resistance determinants [6,7]. Second, colonization is seldom a single-site, single-species event; the same host may carry several organisms across several anatomical niches at once, a polymicrobial, multisite pattern with direct implications for how thoroughly and in what way patients should be screened before invasive procedures. In settings such as Egypt, where the great majority of antibiotics are dispensed without prescription, this largely unmonitored community exposure intensifies the selective pressure driving both phenomena [8,9].

Patients with cardiovascular disease are an instructive and clinically consequential population in which to examine these questions. Repeated healthcare contact, vascular and cardiac interventions, implanted devices and frequent antibiotic courses place them at the interface between the community and the hospital, and resistant infections in this group are associated with substantial morbidity [10,11,12]. How large the colonizing reservoir is in ambulatory, non-critically ill cardiovascular patients; what commensal and low-virulence flora accompanies it; and how frequently carriage spans multiple body sites in the same individual remain largely unquantified.

We therefore screened cardiovascular outpatients at three anatomical sites, with a particular focus on the colonizing and commensal flora and its relationship to community antibiotic use. Specifically, we set out to (i) quantify the overall burden of colonization by organisms with reduced antimicrobial susceptibility; (ii) characterize the co-existing resistant commensal or low-virulence flora that is usually overlooked; and (iii) measure the extent of polymicrobial, multisite carriage in this population, within the broader context of widespread, often unsupervised, community antibiotic use. By showing that resistant colonization is already established in ambulatory cardiovascular patients, we aim to inform pre-procedural screening and community antimicrobial stewardship strategies that could protect these patients before any invasive intervention.

## 2. Results

The study cohort consisted of 36 adults with established cardiovascular disease attending outpatient cardiology clinics. The mean age was 55.3 ± 14.4 years (range, 21 to 85 years), and 63.9% were male. Hypertension was the predominant comorbidity (75.0%), followed by diabetes mellitus (41.7%) and heart failure (27.8%). The cohort also represented a population with substantial healthcare exposure, with 55.6% reporting at least one previous hospitalization, 50.0% having undergone prior percutaneous coronary intervention (PCI), and 25.0% receiving antibiotic therapy during the month of specimen collection. These characteristics provide the clinical context for the ecological analysis of bacterial colonization presented below.

### 2.1. Colonization Burden and Anatomical Distribution

**Figure 1:**
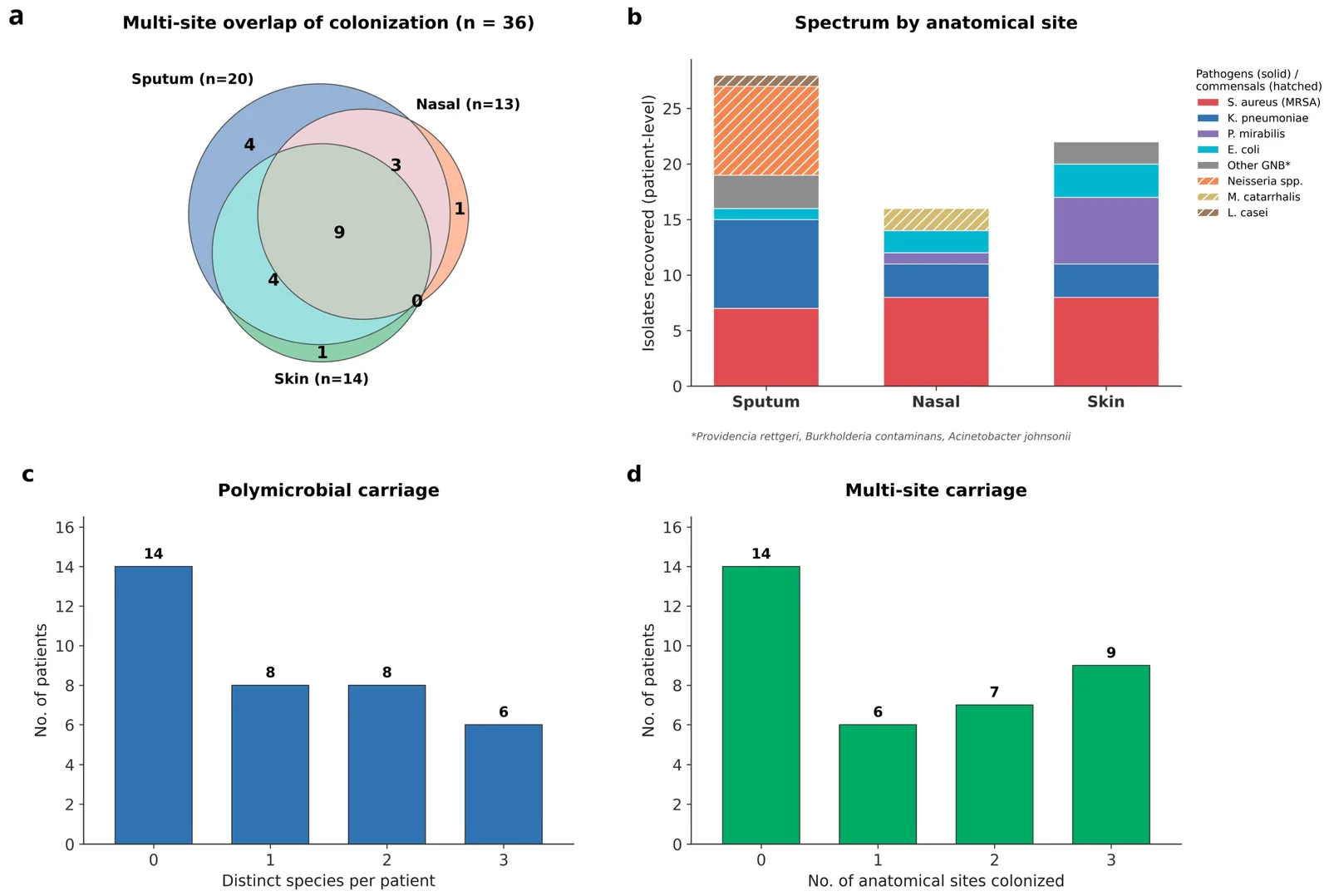
Landscape of bacterial colonization in cardiovascular outpatients (n = 36). (**a**) Three-set overlap of colonization positivity at the sputum, nasal and skin sites; 9 of 22 colonized patients (40.9%) were positive at all three sites and 16 of 22 (72.7%) at two or more sites. (**b**) Spectrum of organisms recovered at each anatomical site (patient-level isolate counts); validated pathogens in solid fill and commensal/low-virulence flora in hatched fill. (**c**) Distribution of the number of distinct species recovered per patient. (**d**) Distribution of the number of anatomical sites colonized per patient. GNB, Gram-negative bacilli.

Overall, colonization by organisms with reduced antimicrobial susceptibility was detected in 22 of the 36 cardiovascular outpatients (61.1%; 95% CI, 44.9 to 75.2) using antimicrobial-supplemented selective media designed to enrich such organisms. Among these, 19 patients (52.8%; 95% CI, 37.0 to 68.0) harboured at least one validated multidrug-resistant (MDR) pathogen, whereas the remaining colonized individuals carried commensal or low-virulence organisms recovered on antimicrobial-containing media, consistent with reduced antimicrobial susceptibility. Colonization prevalence differed significantly across the three anatomical sites (Cochran’s *Q* = 6.62, *p* = 0.037), with sputum demonstrating the highest colonization rate (20/36, 55.6%), followed by skin (14/36, 38.9%) and nasal specimens (13/36, 36.1%) (Table 1; Figure 1b).

**Figure 2:**
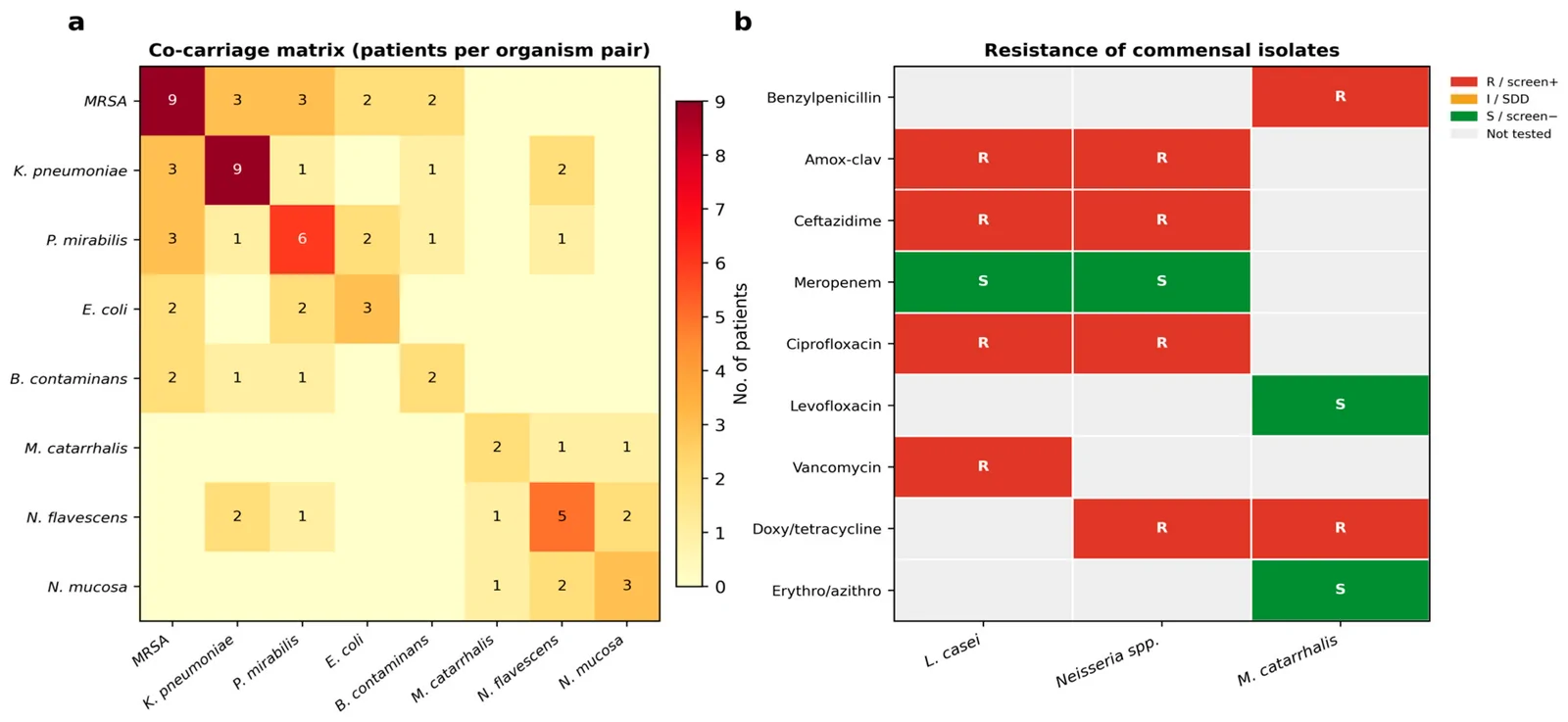
Microbial ecology and commensal resistance. (**a**) Co-carriage matrix showing the number of patients carrying each pair of organisms (diagonal = total carriers of each organism). (**b**) Phenotypic screening results for the commensal and low-virulence isolates (*Lacticaseibacillus casei*, commensal *Neisseria* species and *Moraxella catarrhalis*), shown as a susceptibility heatmap. Because CLSI minimum-inhibitory-concentration breakpoints do not exist for these organisms, results are descriptive screening signals (agar-screen or disc diffusion) rather than validated categories. R, resistant/screen-positive; I, intermediate; S, susceptible/screen-negative.

Validated MDR pathogens and resistant commensal bacteria showed distinct distribution patterns. *Klebsiella pneumoniae* was recovered predominantly from sputum, whereas *Proteus mirabilis* was identified primarily from skin specimens. Methicillin-resistant *Staphylococcus aureus* (MRSA) was detected across all three sites with comparable frequencies. In contrast, commensal *Neisseria* species were recovered exclusively from sputum, while *Moraxella catarrhalis* was confined to nasal specimens. The complete anatomical distribution of all recovered organisms, and the co-carriage of organism pairs, are presented in Table 1 and Table S3 and Figure 2a.

### 2.2. Clinical Correlates of Colonization

To determine whether the collected clinical variables distinguished colonized from non-colonized patients, the 22 colonized and 14 non-colonized individuals were compared across prior hospitalization, prior PCI, concurrent antibiotic therapy and major comorbidities using Fisher’s exact test (Benjamini–Hochberg FDR-adjusted). Prior hospitalization was the strongest correlate of colonization, significant for total colonization (*p* < 0.001) and at every anatomical site; MDR *Klebsiella pneumoniae* and MRSA were recovered exclusively from previously hospitalized patients (nine carriers each; *p* = 0.002). Concurrent antibiotic therapy was also significantly associated with colonization (*p* = 0.006). In contrast, prior PCI, sex, age, diabetes, hypertension, heart failure and smoking showed no significant association (Table 2). Notably, colonization was detected even among patients with no prior hospitalization, indicating that resistant carriage in this population is not confined to those with a documented hospital history.

### 2.3. Multisite Colonization

**Figure 3:**
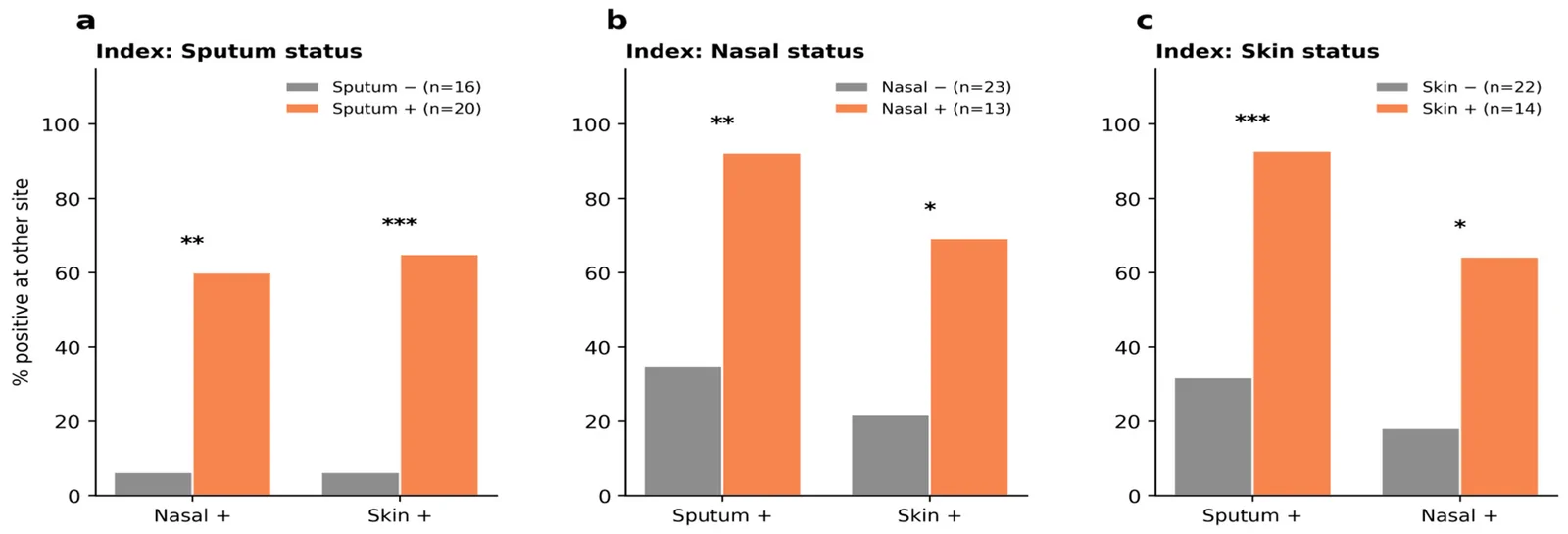
Multisite concordance of carriage. For each index site, namely (**a**) sputum, (**b**) nasal, and (**c**) skin, the percentage of patients positive at the two other sites is shown separately for patients negative (grey) and positive (orange) at the index site. Across all three perspectives, carriage at one site was associated with a markedly higher probability of carriage at the others (Fisher’s exact test), indicating that colonization behaves as a whole-body state rather than a series of independent site events. *** *p* < 0.001, ** *p* < 0.01, and * *p* < 0.05; ns, not significant. Pairwise agreement statistics are given in Supplementary Table S4.

Multisite colonization was common among colonized cardiovascular outpatients. Of the 22 colonized patients, 16 (72.7%) harboured bacteria at two or more anatomical sites, while 9 (40.9%) were colonized simultaneously at all three sampling sites (Figure 1a,d). Colonization showed significant concordance across anatomical sites, with moderate agreement between sputum, nasal and skin specimens (Cohen’s κ = 0.47–0.57; 95% CIs in Table S4). Pairwise site associations, assessed using Fisher’s exact test, were statistically significant for sputum and nasal specimens (*p* = 0.001), sputum and skin specimens (*p* < 0.001), and nasal and skin specimens (*p* = 0.011) (Table S4; Figure 3).

### 2.4. Polymicrobial Colonization

Polymicrobial colonization was identified in a substantial proportion of colonized patients. Among the 22 colonized individuals, 14 (63.6%) harboured two or more distinct bacterial species across the sampled anatomical sites, yielding a total of 42 species-level colonization events and an average of 1.9 bacterial species per colonized patient (Figure 1c; 1.6 pathogenic species among the 19 patients carrying validated MDR pathogens). Co-colonization most frequently involved combinations of validated MDR pathogens, although resistant commensal bacteria were also detected in mixed colonization patterns. The distribution of co-colonizing organism pairs is presented in Figure 2a.

### 2.5. Commensal and Low-Virulence Organisms on Selective Media

Commensal and low-virulence organisms that grew on the antimicrobial-containing screening media were recovered from a substantial proportion of colonized patients. These included *Neisseria flavescens* (five patients), *Neisseria mucosa* (three patients), *Moraxella catarrhalis* (two patients), and *Lacticaseibacillus casei* (one patient). Commensal *Neisseria* species were detected exclusively in sputum specimens, whereas *Moraxella catarrhalis* was recovered only from nasal specimens. Phenotypic testing demonstrated reduced susceptibility to multiple antimicrobial classes including β-lactams, fluoroquinolones, tetracyclines, and glycopeptides, depending on the organism (Figure 2b and Figure S3, Table S5). Because CLSI interpretive breakpoints are unavailable for several of these organisms, their reduced susceptibility is reported descriptively (agar screen and disc diffusion testing) and is not directly comparable to CLSI-categorized resistance; throughout this article, the term “resistant” is reserved for isolates categorized against validated CLSI breakpoints.

## 3. Discussion

To our knowledge, this study is among the first to examine bacterial colonization in ambulatory cardiovascular outpatients from an ecological perspective. In this cohort, organisms with reduced antimicrobial susceptibility were recovered from 61.1% of participants, and validated multidrug-resistant (MDR) pathogens from 52.8%. Colonization was polymicrobial and extended across multiple anatomical sites; 72.7% of colonized patients were positive at two or more sites and 40.9% at all three and included commensal and low-virulence organisms that grew on the antimicrobial-containing screening media. Carriage at one anatomical site was strongly associated with carriage at the others, and prior hospitalization and concurrent antibiotic use were the factors most consistently associated with colonization.

Taken together, these observations indicate that resistant colonization in these patients behaves as a co-occurring, cross-site pattern rather than a series of isolated events, consistent with a whole-body colonization state. That more than half of these non-critically ill outpatients carried MDR bacteria, a burden approaching that reported in inpatient surveillance [5,13] and higher than would be expected outside hospital [14], suggests that the ambulatory cardiovascular clinic is itself part of the community reservoir of antimicrobial resistance. This whole-body colonization state is in keeping with the ability of *Staphylococcus aureus* to establish stable multi-site colonization [15] and with gastrointestinal carriage acting as a reservoir for *Klebsiella pneumoniae* dissemination [16]. This pattern is biologically consistent with the widespread, unsupervised community use of antibiotics reported in Egypt and comparable settings, which suppresses susceptible flora and selects for resistant organisms [8,9]. The parallel recovery of recognized pathogens and commensal or low-virulence organisms on selective media is in line within this framework: naturally transformable commensal *Neisseria* and gut-derived *Enterobacterales* are recognized in the wider literature as potential reservoirs of resistance determinants [6,7], although here, their role is inferred from recovery under antibiotic selection rather than demonstrated genetically.

Several limitations bound these interpretations. As a single-centre, cross-sectional study of 36 patients with sparse cell counts, the analyses are exploratory and hypothesis-generating: the sample size was calculated to estimate the prevalence of MDR colonization, not to detect associations between individual clinical factors and colonization, so because the study was designed primarily for prevalence estimation rather than hypothesis testing, the observed clinical correlates should be regarded as exploratory, and non-significant findings should not be interpreted as evidence of absence of association. The cross-sectional design captures a single time point and cannot establish temporality or causation; in particular, because the exact timing of the last antibiotic dose was not recorded, the association between community antibiotic use and colonization cannot be interpreted causally. Growth on antimicrobial-containing media was treated as a phenotypic screening signal rather than a validated susceptibility category, and for commensal and low-virulence organisms lacking CLSI breakpoints, susceptibility is reported descriptively only. Sputum quality was not scored by Gram stain, so oropharyngeal carry-over cannot be excluded, and no resistance-gene detection or sequencing was performed on the commensal isolates.

Within these limits, the findings carry two practical messages. First, because colonization was common and distributed across multiple sites even among patients without a documented hospital history, single-site sampling is likely to underestimate carriage; this provides a rationale for future studies to evaluate whether pre-procedural screening for resistant bacterial carriage identifies patients at increased risk of subsequent infection, and whether targeted preventive strategies improve clinical outcomes—outcomes which the present cross-sectional study did not measure and cannot demonstrate. Second, the frequent recovery of resistant Gram-negative bacilli from hand (skin) swabs reinforces the importance of routine hand hygiene and appropriate use of personal protective equipment when examining and sampling this population [17,18,19], measures that should be regarded as basic safeguards even in the outpatient setting, where they are often relaxed. In parallel, community-level antimicrobial stewardship is directly relevant to reducing this reservoir at its source, and novel anti-colonization strategies such as antimicrobial peptides derived from previously untapped microbial niches [20] warrant exploration. Larger, longitudinal, multicentre studies incorporating whole-genome sequencing are needed to confirm the genetic relatedness and reservoir role suggested here and to test whether pre-procedural screening and targeted decolonization improve clinical outcomes.

## 4. Materials and Methods

### 4.1. Study Design, Setting and Reporting

This observational, cross-sectional study was conducted at the outpatient cardiology clinics of the Ain Shams Cardio-Thoracic Academy, a tertiary cardiovascular referral centre affiliated with El-Demerdash Hospital, Ain Shams University, Cairo, Egypt, between May and November 2025, with microbiological processing performed at the hospital’s Microbiology Laboratory. Reporting follows the STROBE statement for observational studies [21]. This manuscript is derived from the Master’s thesis of the first author (Y.A.A.), Faculty of Science, Ain Shams University. The analysis focused on the overall colonization burden, the commensal and low-virulence flora, and polymicrobial multisite carriage, using the complete organism set and paired statistical methods suited to the multisite design.

### 4.2. Participants and Sampling

We consecutively enrolled all eligible, consenting adults with established cardiovascular disease who attended the outpatient cardiology clinics during the study window; no random or purposive selection was applied. Eligibility was assessed against the following predefined criteria.

**Inclusion criteria:**

- Age ≥ 18 years;
- Established cardiovascular disease;
- Attendance at the outpatient cardiology clinic during the study period (May–November 2025);
- Provision of written informed consent.

**Exclusion criteria:**

- Incomplete clinical or laboratory records;
- Refusal or inability to provide written informed consent.

Because prior hospitalization, recent antibiotic exposure, active infection and cardiovascular comorbidity were exposures of interest in this study, patients were deliberately not excluded on these grounds. A structured questionnaire captured age, sex, comorbidities, previous hospitalization, prior percutaneous coronary intervention and any antibiotic taken during the month of specimen collection, ascertained through patient and physician reports.

The sample size was calculated to estimate the prevalence of multidrug-resistant bacterial pathogens among cardiovascular patients (the study’s primary outcome) rather than to detect associations between individual clinical variables and colonization; the analyses of clinical correlates are therefore exploratory. The calculation was based on the prevalence of multidrug-resistant bacterial pathogens among cardiovascular patients reported by Ren et al. [11] (0.6–12%), with a 5% alpha error and 80% study power, computed in STATA 10 (StataCorp, College Station, TX, USA), yielding a minimum required sample size of 35 patients; 36 patients were enrolled. It was performed a priori by the Faculty of Medicine statistical unit as a mandatory, documented component of the approved study protocol. From every participant, three specimens were obtained under aseptic conditions, namely an anterior-nares swab, a saline-moistened hand (skin) swab, and an expectorated deep-cough sputum, giving 108 specimens overall, each transported and processed within 30 min. Sputum specimens were collected after oral rinsing by deep coughing into sterile containers; specimen adequacy was assessed to distinguish sputum from saliva, and specimens considered unsuitable for analysis were not accepted. Specimens were processed on the day of collection. No minimum sputum volume was prespecified, and formal Gram-stain-based specimen-quality scoring was not performed.

### 4.3. Isolation, Identification and Susceptibility Testing

Recovery of resistant organisms used antimicrobial-supplemented selective media prepared in the laboratory from commercial dehydrated bases (mannitol salt agar, MacConkey agar and blood agar base; MG, supplied by Science and Technology Center, Giza, Egypt), each used within its labelled shelf-life: cefoxitin mannitol salt agar (cefoxitin 10 mg/L) for methicillin-resistant staphylococci; vancomycin blood agar (vancomycin 8 mg/L, with 5% sheep blood) for vancomycin-non-susceptible Gram-positives; and a MacConkey plate divided into three sectors for resistant Gram-negative bacilli (sector 1, ceftazidime (2 mg/L) + amoxicillin-clavulanate (32 mg/L, amoxicillin component); sector 2, ciprofloxacin (1 mg/L) + doxycycline (4 mg/L); and sector 3, meropenem (1 mg/L)). Bases were reconstituted in distilled water as per the manufacturer’s instructions, and then autoclaved (121 °C, 15 min) and cooled to 45–50 °C before aseptic addition of antibiotic stocks (full lot-preparation details in Table S1). Growth within an antibiotic-containing medium or sector was recorded as a positive phenotypic screen (a signal of non-susceptibility to the corresponding agent), not a definitive susceptibility category.

Selective media were prepared in the laboratory and stored at 4 °C until the following day. Before each sample collection session, six plates from each prepared batch were tested: two uninoculated plates to confirm sterility, and four plates inoculated with previously characterized control isolates routinely used in the laboratory at El-Demerdash Teaching Hospital to confirm performance. The control isolates comprised methicillin-resistant *Staphylococcus aureus*, a susceptible *S. aureus* isolate, multidrug-resistant *Escherichia coli*, and a susceptible *E. coli* isolate; these had been previously identified using BioFire FilmArray system (BioFire Diagnostics, Salt Lake City, UT, USA). All plates were incubated aerobically at 37 °C for 24 h. A batch was accepted only when the uninoculated plates showed no growth and the resistant control isolates grew on the corresponding antimicrobial-containing medium while the susceptible control isolates were inhibited, confirming both the sterility and the discriminatory capacity of each formulation. Formulations showing suboptimal discriminatory performance were excluded, and the selected formulations were verified before use for each collection session. On the day of specimen collection, plates were removed from refrigeration approximately one hour before sampling.

Plates were incubated aerobically at 37 °C for 24–48 h; recovered isolates were sub-cultured on nutrient agar (37 °C, 24 h) to obtain pure colonies. Species-level identification was performed using the VITEK MS system (bioMérieux, Marcy-l’Étoile, France; software version 2025) [22], calibrated for each acquisition group with *Escherichia coli* ATCC 8739; a single-choice confidence value ≥ 60.0% (values > 99% denoting high confidence) was accepted as species-level identification, low-discrimination results (2–4 candidates) were reported at genus level, and absence of a database match was recorded as non-identification. We did not use 16S rRNA gene sequencing for routine identification, as MALDI-TOF provides accurate, reproducible species-level identification for the organisms studied. Susceptibility was determined on the VITEK 2 Compact system (bioMérieux, Marcy-l’Étoile, France) system using the AST-GN (Gram-negative bacilli) and AST-GP (Gram-positive cocci) cards from a 0.50–0.63 McFarland suspension, interpreted per CLSI M100 (35th ed.) [23]. For all isolates with available CLSI interpretive criteria, minimum inhibitory concentrations (MICs) were determined quantitatively on the VITEK 2 system and confirmed the reduced-susceptibility phenotypes first detected on the screening media (representative values in Table S2). Multidrug resistance followed Magiorakos et al.’s definition (acquired non-susceptibility to ≥1 agent in ≥3 categories, excluding intrinsic resistance) [1]. For organisms outside the VITEK 2 repertoire or lacking CLSI breakpoints *Burkholderia contaminans*, commensal *Neisseria* species, *Moraxella catarrhalis* and *Lacticaseibacillus casei*, susceptibility was assessed descriptively using Kirby–Bauer disc diffusion and by growth on the screening media, and is reported without categorical interpretation.

### 4.4. Classification of Pathogens and Commensal Flora

Recovered organisms were classified a priori as validated bacterial pathogens (*S. aureus*, *Klebsiella pneumoniae*, *Escherichia coli*, *Proteus mirabilis*, *Providencia rettgeri*, *Acinetobacter johnsonii* and *Burkholderia contaminans*; the last is a recognized pathogen for which validated CLSI breakpoints are unavailable and whose susceptibility is therefore reported descriptively) or as commensal/low-virulence flora of the respiratory tract and skin (*Neisseria flavescens*, *Neisseria mucosa*, *Moraxella catarrhalis* and *Lacticaseibacillus casei*). This assignment was a clinical ecological classification made from the established literature on each organism’s pathogenic potential and natural habitat and did not itself rely on genotypic testing. Commensal organisms recovered on selective media were characterized descriptively and were not subjected to genotypic resistance-gene testing; their appearance on antimicrobial-supplemented media was interpreted as a phenotypic screening signal of reduced susceptibility rather than confirmed carriage of specific resistance genes.

### 4.5. Statistical Analysis

Data were analyzed in IBM SPSS Statistics v27 (IBM Corp., Armonk, NY, USA) and Python 3 (SciPy, statsmodels). Categorical variables are presented as frequencies and percentages; prevalences are accompanied by Wilson 95% confidence intervals (CIs). Because the three anatomical sites represent repeated observations within the same 36 patients, the overall difference in positivity across sites was tested with Cochran’s *Q*. Pairwise concordance between site pairs was assessed with Fisher’s exact test to determine whether carriage at one site was associated with carriage at another, and the strength of agreement was quantified with Cohen’s κ; the same Fisher’s exact framework underlies the concordance shown in Figure 3. Cohen’s κ is reported with a nonparametric bootstrap 95% CI (5000 resamples). Associations between clinical variables and colonization, and pairwise organism co-occurrence, were assessed with Fisher’s exact test (two-tailed) among factors or organisms present in at least two patients, with odds ratios and 95% CIs estimated by the log-odds-ratio standard-error method (Haldane–Anscombe correction where a zero cell was present) and *p* values adjusted for multiple comparisons with the Benjamini–Hochberg false-discovery-rate procedure (q values); underlying 2 × 2 cell counts, odds ratios, 95% CIs and q values are provided in Tables S3 and S4. Given the sample size and resulting sparse cells, chi-square approximations were avoided, and associations are interpreted as exploratory and hypothesis-generating. A two-sided *p* < 0.05 was considered significant.

## 5. Conclusions

Ambulatory cardiovascular outpatients in Cairo harboured a substantial burden of bacterial colonization characterized by polymicrobial communities, multisite carriage, and a co-existing commensal flora with reduced antimicrobial susceptibility. Together, these findings suggest that colonization in this population reflects a co-occurring, cross-site pattern that may be shaped, at least in part, by widespread unsupervised community antibiotic use. Here, “ambulatory” denotes outpatients not admitted to hospital at the time of sampling; it does not imply the absence of prior hospitalization, which 55.6% reported.

These findings have two important clinical implications. First, because resistant carriage was common even among patients without previous hospitalization, these findings provide a rationale for future studies to evaluate whether targeted screening of the anatomical site planned for invasive cardiac or vascular procedures reduces device- or surgical-site infection; the present cross-sectional design did not follow patients to procedures or infection outcomes and cannot demonstrate that screening prevents infection or is cost-effective. In addition, the frequent recovery of resistant organisms from hand swabs reinforces the importance of routine hand hygiene and appropriate use of personal protective equipment by healthcare workers when managing this patient population. The recovery of reduced-susceptibility commensal flora alongside validated MDR pathogens highlights the potential contribution of the commensal microbiota to the ecology of antimicrobial resistance. These observations further support the need for community antimicrobial stewardship and public awareness regarding the risks associated with unsupervised antibiotic use [24,25]. Larger, longitudinal, multicentre studies incorporating genotypic characterization of commensal organisms are warranted to confirm these findings, clarify their role as potential reservoirs of antimicrobial resistance determinants, and evaluate targeted screening and stewardship strategies in cardiovascular outpatients.

## Figures and Tables

**Table 1:** Spectrum and anatomical distribution of recovered organisms (patient level, n = 36).

Organism	Sputum	Nasal	Skin	Any Site
**Validated MDR pathogens**				
*Staphylococcus aureus (MRSA)*	7 (19.4)	8 (22.2)	8 (22.2)	9 (25.0)
*Klebsiella pneumoniae*	8 (22.2)	3 (8.3)	3 (8.3)	9 (25.0)
*Proteus mirabilis*	0 (0.0)	1 (2.8)	6 (16.7)	6 (16.7)
*Escherichia coli*	1 (2.8)	2 (5.6)	3 (8.3)	3 (8.3)
*Burkholderia contaminans*	1 (2.8)	0 (0.0)	1 (2.8)	2 (5.6)
*Providencia rettgeri*	1 (2.8)	0 (0.0)	1 (2.8)	1 (2.8)
*Acinetobacter johnsonii*	1 (2.8)	0 (0.0)	0 (0.0)	1 (2.8)
**Commensal/low-virulence flora**				
*Neisseria flavescens*	5 (13.9)	0 (0.0)	0 (0.0)	5 (13.9)
*Neisseria mucosa*	3 (8.3)	0 (0.0)	0 (0.0)	3 (8.3)
*Moraxella catarrhalis*	0 (0.0)	2 (5.6)	0 (0.0)	2 (5.6)
*Lacticaseibacillus casei*	1 (2.8)	0 (0.0)	0 (0.0)	1 (2.8)
**Any organism recovered**	**20 (55.6)**	**13 (36.1)**	**14 (38.9)**	**22 (61.1)**

Values are number of patients (%). Per-site counts may exceed the any-site total because a patient may be colonized at more than one site. MDR, multidrug-resistant; MRSA, methicillin-resistant *S. aureus*.

**Table 2:** Clinical correlates of colonization: comparison of colonized (n = 22) and non-colonized (n = 14) patients.

Variable	Non-Colonized (n = 14)	Colonized (n = 22)	*p*
Prior hospitalization	2 (14.3)	18 (81.8)	<0.001
Concurrent antibiotic therapy	0 (0.0)	9 (40.9)	0.006
Prior PCI	9 (64.3)	9 (40.9)	0.305
Male sex	10 (71.4)	13 (59.1)	0.501
Diabetes mellitus	5 (35.7)	10 (45.5)	0.732
Hypertension	11 (78.6)	16 (72.7)	1.000
Heart failure	2 (14.3)	8 (36.4)	0.255
COPD	0 (0.0)	4 (18.2)	0.141
Stroke	1 (7.1)	4 (18.2)	0.628
Smoking	2 (14.3)	6 (27.3)	0.441

Values are number of patients (%) unless otherwise indicated. *p* values were obtained from Fisher’s exact test (two-sided), adjusted for multiple comparisons by the Benjamini–Hochberg procedure. PCI, percutaneous coronary intervention; COPD, chronic obstructive pulmonary disease.

## Data Availability

The original contributions presented in this study are included in the article and Supplementary Materials. Further inquiries can be directed to the corresponding author.

## Supplementary Materials

The following supporting information can be downloaded at https://www.mdpi.com/article/10.3390/antibiotics15090823/s1, Table S1: Preparation of antibiotic stock solutions and final concentrations used for the selective screening media; Table S2: Representative VITEK 2 minimum inhibitory concentrations (MICs) for the validated multidrug-resistant pathogens with available CLSI breakpoints; Table S3: Pairwise bacterial co-occurrence across all participants (Fisher’s exact test), with 2 × 2 cell counts and 95% confidence intervals; Table S4: Concordance of multisite colonization across anatomical sampling sites; Table S5: Phenotypic screening profiles of commensal and low-virulence organisms recovered on antimicrobial-containing selective media; Figure S1: Prevalence of bacterial colonization across anatomical sites with Wilson 95% confidence intervals (n = 36). Point estimates (red) and interval limits (blue) are shown for overall and site-specific positivity and for the principal organisms; Figure S2: Conceptual framework of colonization ecology in cardiovascular outpatients. The schematic summarizes the proposed pathway underlying the study: widespread community antibiotic exposure exerts selection pressure that favours commensal and low-virulence organisms with reduced susceptibility, which coexist with recognized pathogens as polymicrobial, multisite colonization; this colonized state provides a reservoir for potential transmission and, in patients undergoing cardiovascular procedures, contributes to pre-procedural infection risk. The figure illustrates the study hypothesis and does not present new data; Figure S3: Macroscopic colony morphology, microscopic Gram stains, and antimicrobial susceptibility of selected bacterial isolates. (a, b) *N. flavescens*: colony morphology on agar (a) and corresponding microscopic appearance (b). (c, d) *N. mucosa*: colony morphology (c) and microscopic appearance (d). (e, f) *Lacticaseibacillus casei*: colony morphology (e) and microscopic appearance (f). (g, h) *Moraxella catarrhalis*: colony morphology (g) and microscopic appearance (h). (i) Kirby-Bauer disk diffusion for *Moraxella catarrhalis* on blood agar; disks: 1 erythromycin (E, 15 µg), 2 tetracycline (TE, 30 µg), 3 penicillin (P, 10 µg), 4 azithromycin (AZM, 15 µg), 5 levofloxacin (LV, 5 µg). The profile confirms resistance to penicillins and tetracyclines (no zones of inhibition) with susceptibility to macrolides and fluoroquinolones.
