# Supplementary material for: Multisite Colonization Ecology of Multidrug-Resistant and Commensal Bacteria Among Cardiovascular Outpatients: A Cross-Sectional Study from Cairo, Egypt
Source: Antibiotics (Basel). 2026 Aug 25;15(9):823. doi: 10.3390/antibiotics15090823 (PMC13603252; doi:10.3390/antibiotics15090823)
Supplement: Supplementary file 1 [file antibiotics-15-00823-s001.zip › antibiotics-4453930-supplementary.pdf]

Multisite Colonization Ecology of Multidrug-Resistant and Commensal Bacteria among Cardiovascular Outpatients: A Cross-Sectional Study from Cairo, Egypt

Supplementary Materials

Table S1. Preparation of antibiotic stock solutions and final concentrations used for the selective screening media.

Antibiotic	Stock vial	Solvent	Stock conc.	Vol. → 500 mL	Final screening conc.	Reference
Ceftazidime	500 mg	10 mL	50 mg/mL	20 µL	2 mg/L	Wilson & McCabe, 2007
Ciprofloxacin	500 mg	10 mL	50 mg/mL	10 µL	1 mg/L	Lee et al., 2021
Meropenem	500 mg	10 mL	50 mg/mL	10 µL	1 mg/L	CLSI, 2025
Doxycycline	100 mg	1 mL	100 mg/mL	20 µL	4 mg/L	CLSI, 2025
Amoxicillin–clavulanate	625 mg (500/125)	10 mL	50 mg/mL (amox.)	320 µL	32 mg/L (amox.)	CLSI, 2025
Cefoxitin	1000 mg	10 mL	100 mg/mL	50 µL	10 mg/L	Jayaratne & Rutherford, 1999; CLSI, 2025
Vancomycin	500 mg	10 mL	50 mg/mL	80 µL	8 mg/L	Bardet et al., 2017

Ceftazidime, ciprofloxacin, meropenem, doxycycline and amoxicillin–clavulanate were added to the three sectors of the MacConkey plate; cefoxitin to mannitol-salt agar; vancomycin to 5% sheep-blood agar. Bases were autoclaved (121 °C, 15 min) and cooled to 45–50 °C before aseptic addition of the antibiotic stock. Final concentrations were selected from CLSI breakpoints. conc., concentration; Vol., volume; amox., amoxicillin component.

Table S2. Representative VITEK 2 minimum inhibitory concentrations (MICs) for the validated multidrug-resistant pathogens with available CLSI breakpoints.

Organism	Antimicrobial agent	MIC (mg/L)	Interpretation
Methicillin-resistant *Staphylococcus aureus* (MRSA)	Oxacillin	≥ 4	R
	Cefoxitin (screen)	positive	R
	Ciprofloxacin	1	R
	Gentamicin	8	I
	Trimethoprim–sulfamethoxazole	10	R
	Vancomycin	8	I (VISA)
	Linezolid	2	S
Klebsiella pneumoniae	Ceftazidime	≥ 64	R
	Cefepime	4	SDD
	Meropenem	8	R
	Amikacin	16	R
	Ciprofloxacin	1	R
	Trimethoprim–sulfamethoxazole	≥ 20	R
Escherichia coli	Amoxicillin–clavulanate	≥ 32	R
	Ceftazidime	≥ 64	R
	Cefepime	≥ 64	R
	Meropenem	≥ 16	R
	Ciprofloxacin	≥ 4	R
	Trimethoprim–sulfamethoxazole	≥ 320	R
Proteus mirabilis	Ceftazidime	≥ 64	R
	Gentamicin	≥ 16	R
	Ciprofloxacin	2	R
	Doxycycline	≥ 16	R
	Trimethoprim–sulfamethoxazole	≥ 320	R

MICs were determined on the VITEK 2 Compact system (AST-GN card for Gram-negative bacilli; AST-GP card for MRSA) and interpreted per CLSI M100 (35th ed.). Values are representative of the resistant isolates recovered and confirmed the reduced-susceptibility phenotypes first detected on the antimicrobial-supplemented screening media. For MRSA, cefoxitin-screen positivity defines methicillin resistance, and all β-lactams other than anti-MRSA agents are reported as resistant irrespective of individual MICs. The single-isolate MDR organisms not shown (Providencia rettgeri and Acinetobacter johnsonii) displayed comparable multidrug resistance. R, resistant; I, intermediate; SDD, susceptible-dose-dependent; S, susceptible; VISA, vancomycin-intermediate Staphylococcus aureus.

Table S3. Pairwise bacterial co-occurrence across all participants (Fisher's exact test), with 2×2 cell counts and 95% confidence intervals.

Organism 1	Organism 2	Co-carriers (n)	Cells (a/b/c/d)	OR	OR 95% CI	P	q
N. flavescens	*N. mucosa*	2	2/3/1/30	20.00	1.37–291.1	0.045	0.614
MRSA	*B. contaminans*	2	2/7/0/27	18.33	0.79–424.4	0.057	0.614
P. mirabilis	*E. coli*	2	2/4/1/29	14.50	1.06–198.8	0.066	0.614
MRSA	*E. coli*	2	2/7/1/26	7.43	0.59–94.3	0.148	0.756
MRSA	*P. mirabilis*	3	3/6/3/24	4.00	0.64–25.0	0.151	0.756
M. catarrhalis	*N. mucosa*	1	1/1/2/32	16.00	0.71–361.7	0.162	0.756
M. catarrhalis	*N. flavescens*	1	1/1/4/30	7.50	0.39–145.0	0.262	0.963
P. mirabilis	*B. contaminans*	1	1/5/1/29	5.80	0.31–108.6	0.310	0.963
K. pneumoniae	*B. contaminans*	1	1/8/1/26	3.25	0.18–58.1	0.443	1.000
K. pneumoniae	*N. flavescens*	2	2/7/3/24	2.29	0.32–16.5	0.581	1.000
MRSA	*K. pneumoniae*	3	3/6/6/21	1.75	0.33–9.2	0.660	1.000
K. pneumoniae	*P. mirabilis*	1	1/8/5/22	0.55	0.06–5.5	1.000	1.000
P. mirabilis	*N. flavescens*	1	1/5/4/26	1.30	0.12–14.2	1.000	1.000

Pairs are ordered by P value. Cells a/b/c/d denote both-positive / organism-1-only / organism-2-only / neither. Odds ratios (OR) are from 2×2 tables (Haldane–Anscombe correction applied where a zero cell was present); 95% CIs by the log-OR standard-error method; P from Fisher's exact test; q from the Benjamini–Hochberg false-discovery-rate procedure across all organism pairs tested. No co-occurrence remained significant after FDR correction; the strongest signals were between the two commensal Neisseria species and between Proteus mirabilis and Escherichia coli (both skin-associated). MRSA, methicillin-resistant Staphylococcus aureus.

Table S4. Concordance of multisite colonization across anatomical sampling sites.

(a) Distribution of the number of anatomical sites colonized among the 22 colonized patients.

Number of anatomical sites colonized	n (%)
One site	6 (27.3)
Two sites	7 (31.8)
Three sites	9 (40.9)

(b) Pairwise site concordance with 2×2 cell counts, Cohen's κ, odds ratio, and adjusted significance.

Site pair	Cells (both/A-only/B-only/neither)	κ (95% CI)	OR (95% CI)	P	q
Sputum–Nasal	12 / 8 / 1 / 15	0.51 (0.26–0.78)	22.5 (2.5–205.8)	0.001	0.002
Sputum–Skin	13 / 7 / 1 / 15	0.57 (0.31–0.83)	27.9 (3.0–257.3)	<0.001	0.001
Nasal–Skin	9 / 4 / 5 / 18	0.47 (0.15–0.75)	8.1 (1.7–37.8)	0.011	0.011

For each pair, site A and site B follow the pair label (e.g., for Sputum–Nasal, A = sputum, B = nasal). Overall positivity differed across the three sites (Cochran's Q = 6.62, P = 0.037). Fisher's exact test was used for association (concordance) between site pairs; Cohen's κ quantifies agreement (95% CI by nonparametric bootstrap, 5,000 resamples); odds-ratio 95% CIs by the log-OR standard-error method; q by the Benjamini–Hochberg procedure. This table corresponds to Figure 3 of the main manuscript.

Table S5. Phenotypic screening profiles of commensal and low-virulence organisms recovered on antimicrobial-containing selective media.

Agent	Class	*L. casei*	*Neisseria* spp.	*M. catarrhalis*
Benzylpenicillin	Penicillin	–	–	R
Amoxicillin–clavulanate	Aminopenicillin + BLI	R	R	–
Ceftazidime	Cephalosporin (3rd)	R	R	–
Meropenem	Carbapenem	S	S	–

Agent	Class	*L. casei*	*Neisseria* spp.	*M. catarrhalis*
Ciprofloxacin	Fluoroquinolone	R	R	–
Levofloxacin	Fluoroquinolone	–	–	S
Vancomycin	Glycopeptide	R	–	–
Doxycycline / tetracycline	Tetracycline	–	R	R
Erythromycin / azithromycin	Macrolide	–	–	S

R, resistant or agar-screen positive; S, susceptible or screen-negative; –, not tested. Results are descriptive (agar-screen or disk diffusion), as CLSI minimum-inhibitory-concentration breakpoints do not exist for commensal Neisseria (N. flavescens / N. mucosa; M100 covers only N. gonorrhoeae and N. meningitidis) or the Burkholderia cepacia complex. Neisseria spp. denotes N. flavescens and N. mucosa. BLI, β-lactamase inhibitor.

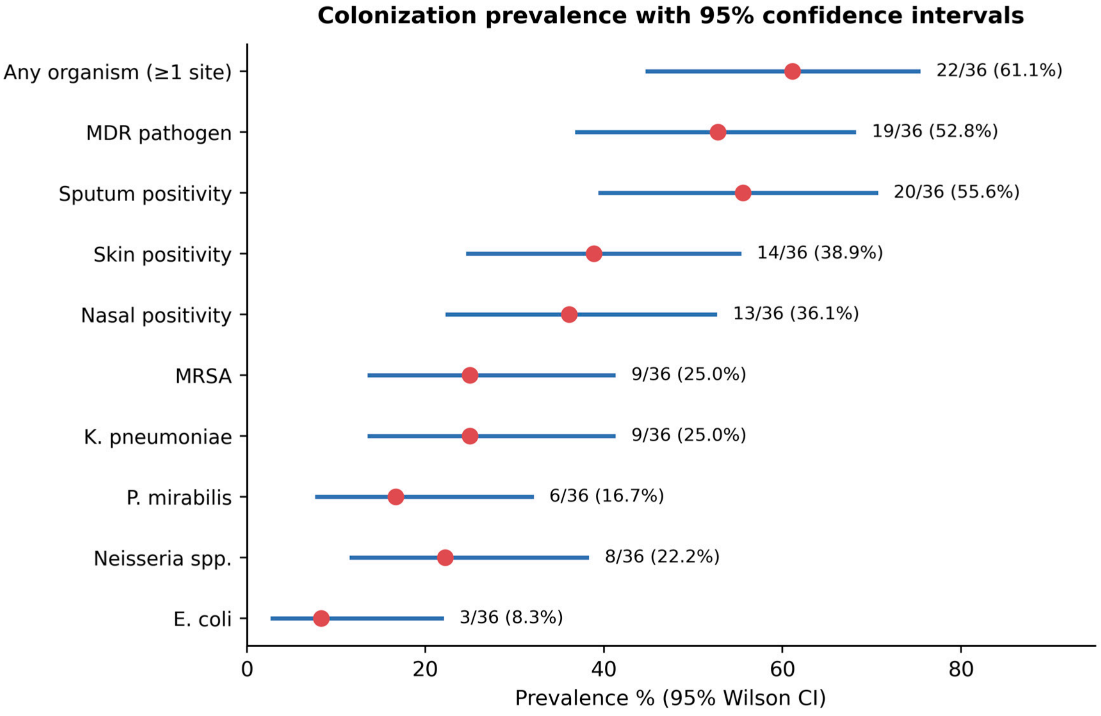

Figure S1. Prevalence of bacterial colonization across anatomical sites with Wilson 95% confidence intervals (n = 36). Point estimates (red) and interval limits (blue) are shown for overall and site-specific positivity and for the principal organisms.

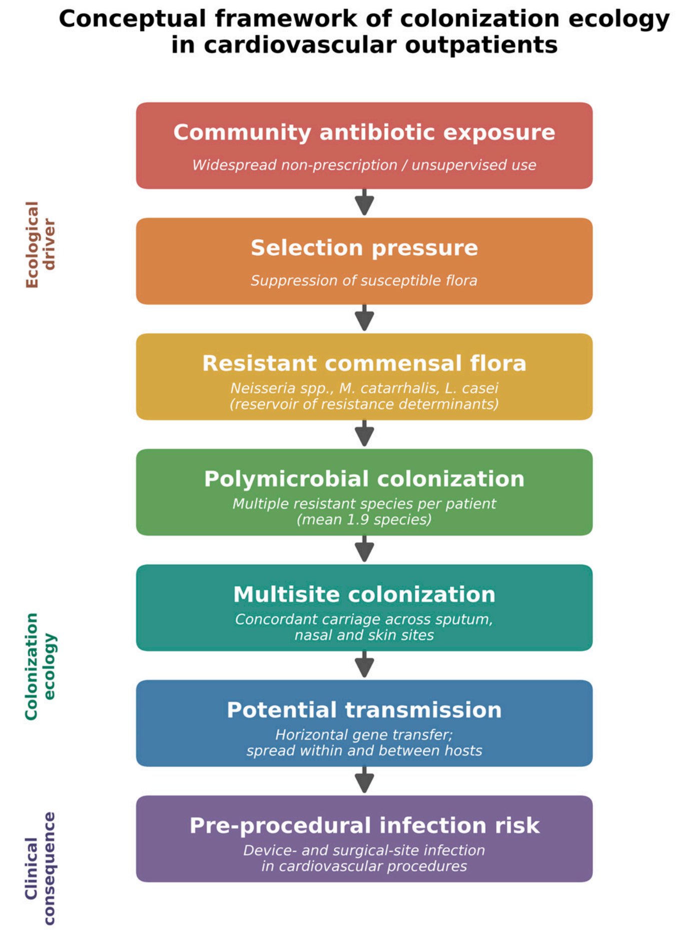

Figure S2. Conceptual framework of colonization ecology in cardiovascular outpatients. The schematic summarizes the proposed pathway underlying the study: widespread community antibiotic exposure exerts selection pressure that favours commensal and low-virulence organisms with reduced susceptibility, which coexist with recognized pathogens as polymicrobial, multisite colonization; this colonized state provides a reservoir for potential transmission and, in patients undergoing cardiovascular procedures, contributes to pre-procedural infection risk. The figure illustrates the study hypothesis and does not present new data.

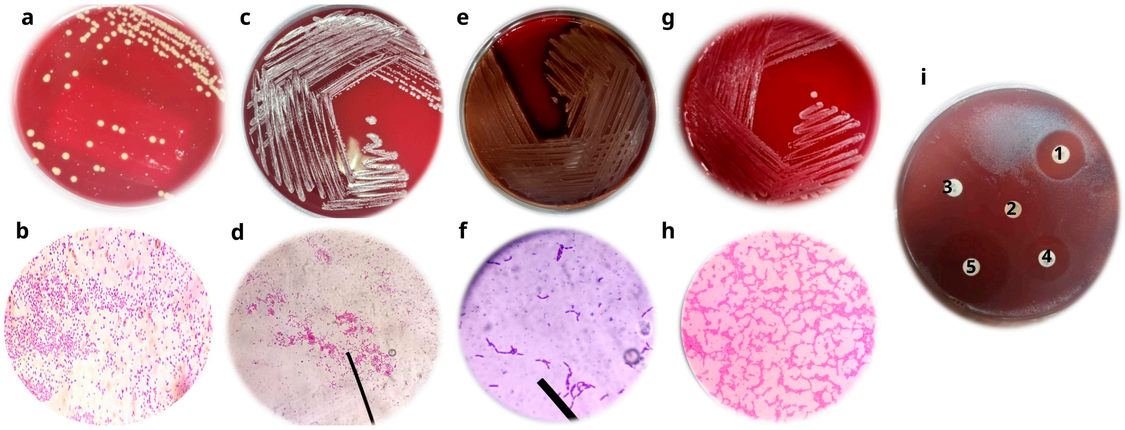

Figure S3. Macroscopic colony morphology, microscopic Gram stains, and antimicrobial susceptibility of selected bacterial isolates. (a, b) *N. flavescens*: colony morphology on agar (a) and corresponding microscopic appearance (b). (c, d) *N. mucosa*: colony morphology (c) and microscopic appearance (d). (e, f) *Lacticaseibacillus casei*: colony morphology (e) and microscopic appearance (f). (g, h) *Moraxella catarrhalis*: colony morphology (g) and microscopic appearance (h). (i) Kirby-Bauer disk diffusion for *Moraxella catarrhalis* on blood agar; disks: 1 erythromycin (E, 15 µg), 2 tetracycline (TE, 30 µg), 3 penicillin (P, 10 µg), 4 azithromycin (AZM, 15 µg), 5 levofloxacin (LV, 5 µg). The profile confirms resistance to penicillins and tetracyclines (no zones of inhibition) with susceptibility to macrolides and fluoroquinolones.
